# Effects of Repetitive Transcranial Magnetic Stimulation in Patients With Mild Cognitive Impairment: A Meta-Analysis of Randomized Controlled Trials

**DOI:** 10.3389/fnhum.2021.723715

**Published:** 2021-10-26

**Authors:** Xinqi Zhang, Xiaoyong Lan, Chanjuan Chen, Huixia Ren, Yi Guo

**Affiliations:** ^1^Department of Neurology, Shenzhen People's Hospital (The First Affiliated Hospital of Southern University of Science and Technology, The Second Clinical Medical College of Jinan University), Shenzhen, China; ^2^Department of Neurology, The Second Clinical Medical College, Jinan University (Shenzhen People's Hospital), Shenzhen, China; ^3^The First Affiliated Hospital, Jinan University, Guangzhou, China

**Keywords:** mild cognitive impairment, repetitive transcranial magnetic stimulation, intervention, memory, treatment, therapy, cognitive function

## Abstract

**Background:** Mild cognitive impairment (MCI) is an intermediary state between normal aging and dementia. It has a high risk of progression in patients with Alzheimer's disease (AD). Repetitive transcranial magnetic stimulation (rTMS) is a non-invasive brain stimulation technique used to improve cognitive deficits in patients with MCI and AD. Although previous meta-analyses included studies carried on patients with MCI and AD, few studies have analyzed patients with MCI independently. This meta-analysis aimed to evaluate the effects and safety of rTMS on cognition function in patients with MCI and factors that may influence such effects.

**Methods:** Data used in this study were searched and screened from different databases, including PubMed, Web of Science, Embase, the Cochrane Library, Chinese National Knowledge Infrastructure (CNKI), Chinese Technical Periodicals (VIP), Wanfang Database, and China BioMedical Literature Database (SinoMed). The retrieved studies were carefully reviewed, data were extracted, and the quality of data was assessed.

**Results:** A total of 12 studies involving 329 patients with MCI were included in the present meta-analysis. The analyses results revealed that rTMS improved cognitive function [standardized mean difference (SMD) = 0.83, 95% confidence interval (CI) = 0.44–1.22, *p* = 0.0009] and memory function (SMD = 0.73, 95% CI = 0.48–0.97, *p* < 0.00001) in the MCI + rTMS active group when compared to the sham stimulation group. The showed that: (1) cognitive improvement was more pronounced under high-frequency rTMS stimulation of multiple sites, such as the bilateral dorsolateral prefrontal cortex and (2) more than 10 rTMS stimulation sessions produced higher improvement on cognition function in patients with MCI.

**Conclusions:** This study shows that rTMS can improve cognitive function in patients with MCI, especially when applied at high frequency, multi-site, and for a prolonged period. However, further studies are required to validate these findings and explore more effective stimulation protocols and targets.

**Systematic Review Registration:** [http://www.crd.york.ac.uk/PROSPERO/], identifier: CRD 42021238708.

## Introduction

Mild cognitive impairment (MCI) is under the diagnostic criteria for dementia, which shows progressive impairment in memory or other cognitive functions that do not influence an individual's daily life (American Psychiatric Association, 2013). Mild cognitive impairment is an intermediary state between normal aging and dementia. For people aged 65 years and above, the prevalence of MCI is ~16–20% (Petersen, 2004; Roberts and Knopman, 2013). And ~10–15% of patients with MCI convert to dementia annually (Mitchell and Shiri-Feshki, 2009; Roberts and Knopman, 2013).

Clinical data show that the currently used drugs cannot effectively alleviate MCI symptoms (Petersen et al., 2018). Cholinesterase inhibitors, which are used to treat Alzheimer's disease (AD), were used to treat MCI (Birks and Flicker, 2006). Their clinical effects were unsatisfactory (Doody et al., 2009) and the adverse effects of the drugs have been more pronounced than the improvement of cognitive function (Cooper et al., 2013; Tricco et al., 2013; Fitzpatrick-Lewis et al., 2015). Amyloid and tau are considered important treatment targets in AD. Aducanumab, which was recently approved by the FDA, but this approval has been intensely debated. Researchers were concerned that the approval was premature, given conflicting evidence regarding aducanumab's clinical efficacy from two phases 3 randomized clinical trials (RCTs). Clinical trials showed that high-dose of aducanumab caused vasogenic edema and cortical microhemorrhage in about 40% of patients (Haeberlein et al., 2020). Although these complications were safely managed in the trials but also raised issues about the clinical safety of aducanumab (Haeberlein et al., 2020). Existing clinical guidelines do not recommend any specific drug for the treatment of MCI. Therefore, researchers have attempted to develop non-pharmacological interventions for MCI.

In recent years, non-invasive brain stimulation has attracted much attention from researchers. Transcranial magnetic stimulation (TMS) is a technique that delivers strong magnetic pulses to the brain regions to induce electrical currents that non-invasively stimulate and modulate the cerebral cortex (Ni and Chen, 2015; Rabey and Dobronevsky, 2016). Repetitive transcranial magnetic stimulation (rTMS) is stimulation in which multiple pulses are continuously emitted at the same frequency to excite or inhibit cortical function specifically. The specific stimulation parameters of rTMS depend on the purpose of treatment or research. The effects of rTMS on the excitability of cortex are frequency-dependent. High and low-frequency stimulation increases and decreases the excitability of the cerebral cortex, respectively (Huang et al., 2005). Thus, by changing the stimulation frequency, rTMS can bidirectionally regulate the balance between excitation and inhibition in the cortex, thereby improving brain function. Disruption of the balance between excitation and inhibition is generally thought to promote the pathogenesis of AD (Weiler et al., 2020). Accordingly, the benefits of rTMS on dementia and cognitive impairment have been investigated (Rutherford et al., 2015; Padala et al., 2018).

Several meta-analyses have summarized the effects of rTMS on cognitive impairment in patients with AD (Lin et al., 2019; Chou et al., 2020; Wang et al., 2020). The studies included patients with MCI and AD in most previous meta-analyses, and few studies analyzed patients with MCI independently (Chou et al., 2020). A recent meta-analysis reported the effect of rTMS on cognition in patients with MCI (Jiang et al., 2020), but the studies included were not exclusively randomized controlled trials (RCTs). Furthermore, different interventions were used as controls in some of the included studies, indicating high heterogeneity across the studies.

Although rTMS has been reported to improve MCI, the optimal stimulation protocols and application parameters are not well-understood. Various rTMS stimulation protocols have been reported in the previous studies, such as combinations of sites, frequency, intensity, number of stimuli, and number of treatments. The current meta-analysis was based on strict inclusion criteria, carried out on newly published RCTs. The pooled effects of rTMS were analyzed to provide the updated evidence regarding the effects and safety of rTMS on cognitive function in patients with MCI.

## Materials and Methods

The present meta-analysis was conducted in line with the guidelines of the Preferred Reporting Items for Systematic Reviews and Meta-Analyses (PRISMA) (Page et al., 2021). The study was registered in the open-access database, International Prospective Register of Systematic Reviews (PROSPERO) (http://www.crd.york.ac.uk/PROSPERO/; registration number: CRD 42021238708).

### Search Strategies

The following databases were searched to identify studies published before March 11, 2020: PubMed, Web of Science, Embase, the Cochrane Library, Chinese National Knowledge Infrastructure (CNKI), Chinese Technical Periodicals (VIP), Wanfang Database, and China BioMedical Literature Database (SinoMed). The search was performed using the following English keywords: (“Cognitive Dysfunction” OR “mild cognitive impairment” OR “MCI”) AND (“Transcranial Magnetic Stimulation” OR “TMS” OR “repetitive transcranial magnetic stimulation” OR “rTMS”), In addition, the following Chinese keywords were used: (“qingdurenzhizhangai” OR “qingdurenzhisunhai” OR “轻度认知障碍” OR “轻度认知损害”) AND (“chongfujingluciciji” OR “重复经颅磁刺激”). The reference lists of identified articles were manually searched to select other potential studies.

### Inclusion and Exclusion Criteria

Studies that met the following criteria were included in the present meta-analysis: (1) randomized controlled studies investigating the effects of rTMS on cognitive function in patients with MCI; (2) participants diagnosed with MCI based on any diagnostic criteria, such as the Petersen criteria (Petersen et al., 1999), criteria for MCI due to AD by the National Institute on Aging-Alzheimer's Association (Albert et al., 2011), or the fifth edition of the Diagnostic and Statistical Manual of Mental Disorders; (3) the experimental group received rTMS treatment; (4) the control group received sham rTMS stimulation; (5) outcomes included global cognitive ability and specific cognitive domains as determined by neuropsychological tests or other objective measures.

The exclusion criteria were as follows: (a) animal research; (b) severe degree of cognitive impairment; (c) articles were reviews or conference reports; (d) articles published in the form of case reports, commentaries, and letters; (e) studies that did not have sham stimulation as the control group.

The identified studies were initially screened by reading the title and abstract. Those that did not meet the inclusion criteria were excluded. In case of ambiguity, the entire article was read to determine eligibility.

### Data Extraction

Two researchers working independently examined and extracted data from the selected studies. The information extracted included: (1) general characteristics: first author, year of publication, number of participants, mean age, sex ratio, and baseline cognitive or memory score (MMSE or MOCA); (2) study design, selection criteria, study duration, and outcome indicators; and (3) treatment intervention: stimulation location, intensity, frequency, number of treatments, total pulses per treatment, sham stimulation method, dropout rate, and adverse effects. If the data from the same study appears in more than one database, the one with the highest number of patients and detailed information was selected. If outcomes were reported at different time points, the data was collected immediately after selecting the interventions. Corresponding authors were contacted if the study provided insufficient data or unclear information. Any discrepancies in data obtained by the two researchers were resolved by discussing with another professional a third researcher to reach a consensus.

### Quality of Studies and Risk of Bias

The quality of included studies was evaluated using the criteria described in the Cochrane Handbook for Systematic Reviews of Interventions (Cumpston et al., 2019). Two researchers (LXY and CCJ) independently extracted data from each included study and compared the results. Any discrepancies in results obtained by the two researchers were discussed, and a consensus was amicably reached.

The risk of bias was assessed using methods recommended by the Cochrane Collaboration Network (Cumpston et al., 2019), and the following characteristics were assessed: (a) adequacy of sequence generation; (b) allocation concealment; (c) use of blinding; (d) method of handling incomplete outcome data (shedding); (e) evidence of selective outcome reporting; and (f) other potential risks that could compromise the validity of the study. The risk of bias for each domain was categorized as low, high, or unclear.

### Statistical Analysis

The effect of rTMS on cognitive function in patients with MCI was defined as the mean difference in the change of cognitive indicators relative to baseline (pre-stimulus scale scores) in the experimental and control groups. Considering the diversity of cognitive indicators applied in the included studies, standardized mean differences (SMDs) and 95% confidence intervals (CIs) were used to summarize eligible trial pooled effect sizes. The pre-and post-stimulus scale scores for each group were recorded. The following formulas were used for studies that did not show a net change in cognitive indicator scores:


$$\begin{array}{l} \text{Mean change}=\text{Mean final}-\text{Mean baseline};\\ \text{ SD change}=\sqrt{{\text{SD baseline}}^{2}+{\text{SD final}}^{2}-(2\times \text{coefficent}}\\ \;\times \text{ SD baseline}\times \text{SD final}) \end{array}$$

For the studies in which raw data were presented as mean ± standard error, standard deviation (SD) was calculated using the following formula:


$$\begin{array}{l} \text{SE}=\text{SD}/\sqrt{n}\;\left(\text{n indicates the number of participants}\right). \end{array}$$

Data obtained in this study were integrated using Cochrane Review Manager (RevMan) 5.4 software (Cochrane Collaboration Network Review Manager). Heterogeneity between included trials was quantified using the *I*^2^ statistic, and the values >50% were considered significant heterogeneity. A random-effects model was used to obtain reliable outcomes since heterogeneity existed among the studies. Funnel plots were constructed, and Begg's and Egger's tests were performed to evaluate publication bias (Egger et al., 1997). Statistical analyses were performed using STATA 16.0 software (STATA Corp., College Station, TX, USA).

### Subgroup Analyses

To further investigate the factors influencing the effects of rTMS on cognitive outcomes, the following four subgroup analyses were conducted: (1) effects on global cognition, (2) effects on memory, (3) stimulation sites (single site vs. multiple sites), and (4) number of treatments (≤10 vs. >10 treatment sessions).

## Results

### Search Results

**Figure 1:**
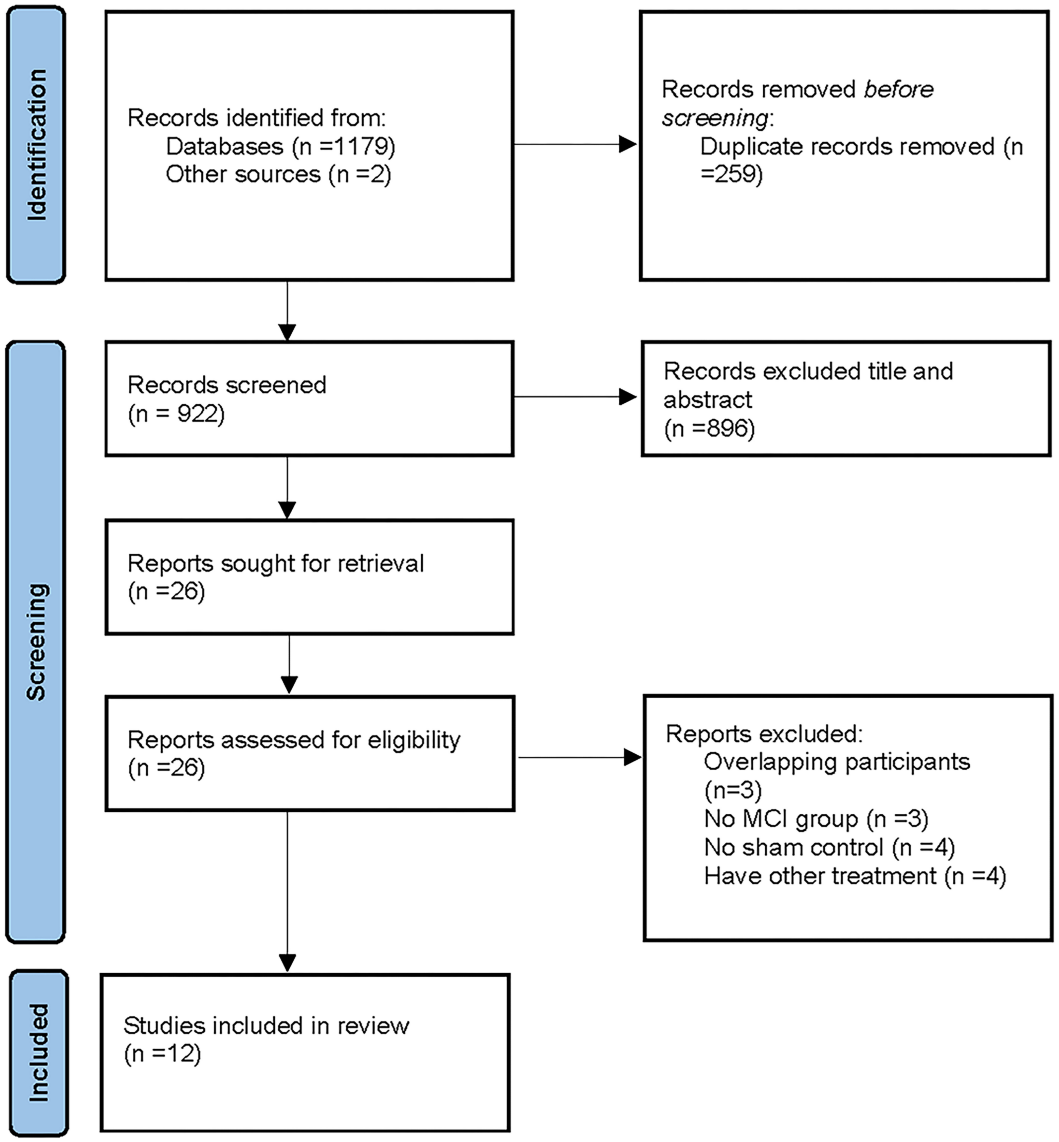
A flow diagram showing the search and inclusion criteria for studies [Based on PRISMA statement (www.prisma-statement.org)]. Other sources from Chou et al. (2020).

A total of 1,179 studies were retrieved from the selected databases, and two studies were obtained from the reference lists of identified studies. Next, 259 duplicate studies were removed, and the remaining 922 studies were further assessed for eligibility. A total of 873 studies were excluded after reading their titles and abstracts. In addition, 58 articles were excluded after full-text reading. Eventually, 12 studies were included in the present meta-analysis. The screening and selection process is illustrated in Figure 1.

### Study Characteristics

Twelve studies were included in the present meta-analysis. Seven were written in English, and five studies in Chinese, with 329 patients with MCI. The baseline characteristics of the included studies are presented in Table 1. The number of participants randomly assigned to the active rTMS group was 182, while those randomly assigned to the sham rTMS group were 178. Only one study used low-frequency rTMS (1 Hz) (Turriziani et al., 2012); the remaining studies used high-frequency rTMS [5 Hz (Solé-Padullés et al., 2006), 10 Hz (Drumond Marra et al., 2015; Long et al., 2018; Padala et al., 2018; Wen et al., 2018; Cui et al., 2019; Yuan et al., 2021) 15 Hz (Deng et al., 2019), and 20 Hz (Han et al., 2013; Yang et al., 2014; Koch et al., 2018)]. The rTMS stimulation sites included the left dorsolateral prefrontal cortex (DLPFC), the right DLPFC, the bilateral DLPFC, and the left precuneus. The stimulation intensity was 80–120% of the resting motor threshold (MT). The number of treatments ranged from 1 to 40 sessions. Similar stimulation parameters were applied in the control group, except that the coils were placed vertically or a special sham stimulation coil was used instead.

**Table 1:** Characteristics of studies included in the meta-analysis.

**References**	**Diagnostic criteria for MCI**	**Study design**	**Interventions**	**Age (M + SD)**	**Sample size (M/F)**	**Site for stimulation**	**Stimulation frequency Stimulation intensity (%MT)**	**Treatment frequency, number of pluses each time**	**Cognitive outcomes/measure**	**Sham rTMS**
Solé-Padullés et al. (2006)	Diagnostic and Statistical Manual fourth edition criteria	Parallel	T: active rTMS	66.95 (9.43)	20 (5/15)	Left DLPFC	5 HZ	1 time	Memory: Associative memory assessment	Tilted coil
			C: sham rTMS	68.68 (7.78)	19 (6/13)		80%	500 pulses		
Turriziani et al. (2012)	Petersen Diagnostic Criteria	cross-over	T: active rTMS	66.4 (5.7)	8 (6/2)	Bilateral DLPFC	1 HZ	1 time	Memory: Non-verbal recognition memory task	Tilted coil
			C: sham rTMS				90%	600 pulses		
Han et al. (2013)	Petersen Diagnostic Criteria	Parallel	T: active rTMS	66.5 (5.02)	22 (8/14)	Bilateral DLPFC	20 HZ	40 times	Global cognitive function: MoCA; Memory: associative learning test, episodic memory test; Executive function and attention: TMT-A, WCST, VFT, DSST	Tilted coil
			C: sham rTMS	66.7 (5.25)	18 (6/12)		80%	36,000 pulses		
Yang et al. (2014)	Petersen Diagnostic Criteria	Parallel	T: active rTMS	66 (6)	18 (8/10)	Bilateral DLPFC	20 HZ	40 times	Global cognitive function: MMSE	Inactive coil with sound
			C: sham rTMS	66 (7)	15 (7/8)		80%	36,000 pulses		
Drumond Marra et al. (2015)	Clinical/ neuropsychological criteria for MCI	Parallel	T: active rTMS	65.1 (3.5)	15 (6/9)	Left DLPFC	10 Hz	10 times	Memory: RBMT, WMS, WAIS-III; Executive function and attention: TMT-B, VFT	Sham coil
			C: sham rTMS	65.2 (4.1)	19 (6/13)		110%	2,000 pulses		
Koch et al. (2018)	Prodromal AD (Dubois et al., 2016)	cross-over	T: active rTMS	70 (5)	14 (7/7)	L parietal region (precuneus)	20 Hz	10 times	Global cognitive function: MMSE; Memory: RAVLT; Executive function: Frontal Assessment Battery (FAB); Attention: DSST	Sham coil
			C: sham rTMS				100%	1,600 pulses		
Padala et al. (2018)	Petersen Diagnostic Criteria	cross-over	T: active rTMS	65.6 (9.3)	9 (8/1)	Left DLPFC	10 Hz	10 times	Global cognitive function: MMSE; Executive function: TMT	Tilted coil
			C: sham rTMS				120%	3,000 pulses		
Wen et al. (2018)	American Neurological Association Quality Standards Branch (QSSAAN) MCI diagnostic criteria	Parallel	T: active rTMS	64.17 (5.21)	23 (14/9)	Left DLPFC	10 Hz	20 times	Global cognitive function: MoCA; Memory: RBMT	Tilted coil
			C: sham rTMS	65.91 (4.93)	20 (10/12)		80%	400 pulses		
Long et al. (2018)	Petersen Diagnostic Criteria	Parallel	T: active rTMS	68.27 (9.85)	15 (8/7)	Left DLPFC	10 Hz	10 times	Global cognitive function: MoCA; Memory: CMS	Sham coil
			C: sham rTMS	65.63 (9.36)	15 (6/9)		90%	1,000 pulses		
Cui et al. (2019)	NIA-AA criteria (2011) for “MCI due to AD”	Parallel	T: active rTMS	73.91 (10.01)	11 (3/8)	Right DLPFC	10 Hz	10 times	Global cognitive function: MMSE, ACE-III; Memory: logic memory test, AVLT; Executive function and attention: TMT-A, TMT-B	Tilted coil
			C: sham rTMS	74.00 (7.62)	10 (5/5)		90%	1,500 pulses		
Deng et al. (2019)	Expert consensus on prevention and treatment of cognitive dysfunction in China	Parallel	T: active rTMS	67.20 (5.89)	25 (15/10)	Left DLPFC	15 Hz	15 times	Global cognitive function: MoCA;	Tilted coil
			C: sham rTMS	68.30 (5.60)	25 (14/11)		80%	900 pulses		
Yuan et al. (2021)	Petersen Diagnostic Criteria	Parallel	T: active rTMS	65.08 (4.89)	12 (6/6)	Left DLPFC	10 Hz	20 times	Global cognitive function: MoCA;	Tilted coil
			C: sham rTMS	64.67 (4.77)	12 (5/7)		80%	400 pulses		

*ACE-III, Addenbrooke's Cognitive Examination-III; AVLT, auditory verbal learning test; CMS, Clinical Memory Scale; DLPFC, Dorsolateral Prefrontal Cortex; DSST, Digit Symbol Substitution Test; F, female; M, male; M, mean; MT, Motor Threshold; MMSE, Mini-Mental State Examination; MoCA, Montreal Cognitive Assessment; RBMT, Rivermead Behavioral Memory Test; rTMS, repetitive Transcranial Magnetic Stimulation; SD, Standard deviation; TMT, Trail Making Test; VFT, Verbal Fluency Test; WAIS-III, Wechsler Adult Intelligence Scale III; WCST: Wisconsin Card Sorting Test; WMS, Wechsler Memory Scale*.

Concerning outcome indicators, overall cognitive function was assessed using the Montreal Cognitive Assessment (MoCA) scale and the Mini-Mental State Examination (MMSE). Further, the memory function was assessed using the Associative Learning Test, the Rivermead Behavioral Memory Test (RBMT), the Clinical Memory Scale (CMS), and the Logical Memory Test. Executive functions and attention were assessed using the Trail Making Test A (TMT-A) and B (TMT-B) and Verbal Fluency Test (VFT) (Table 1).

### Study Quality

**Figure 2:**
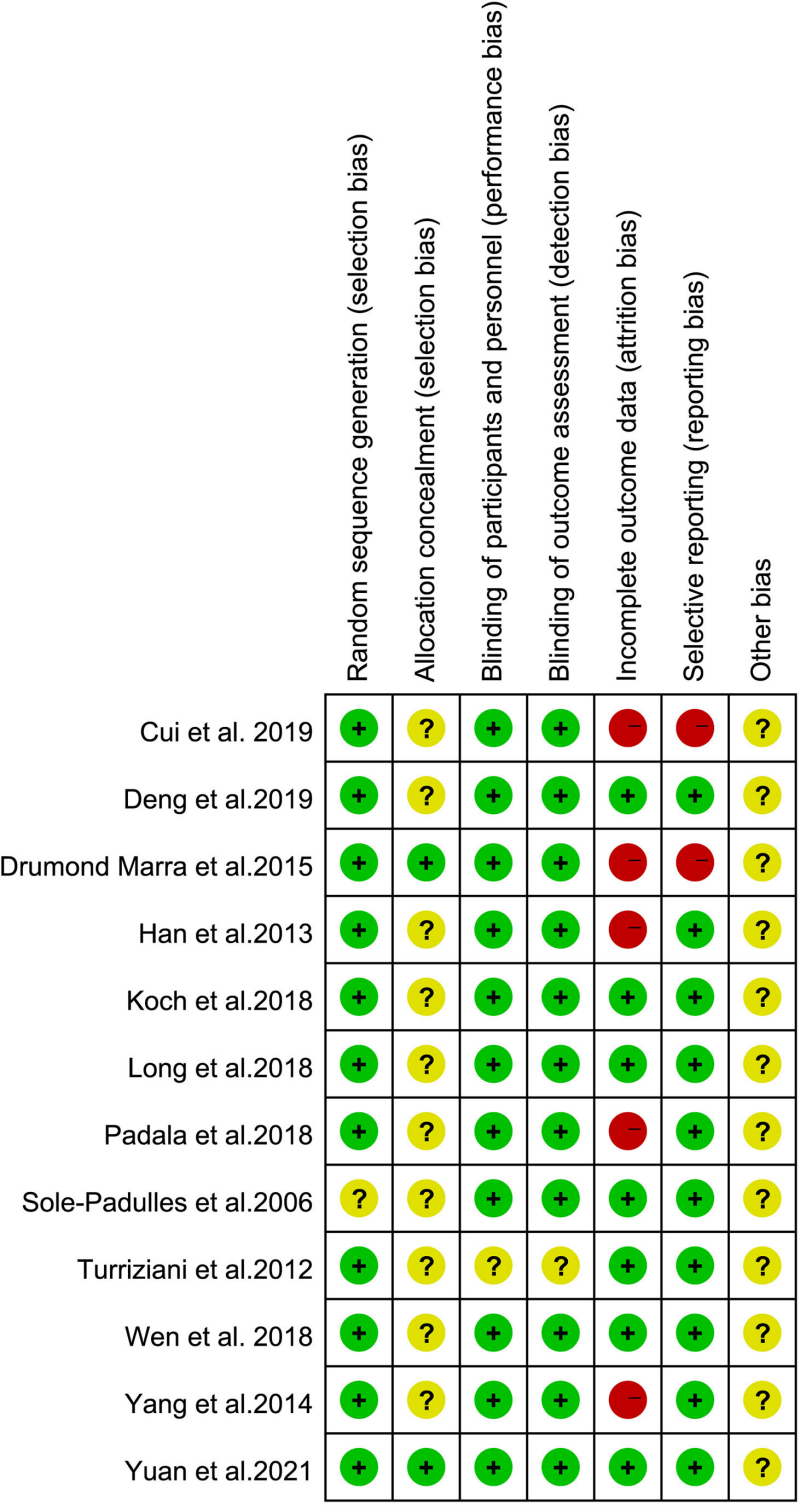
Risk of bias summary: review authors' judgments about each risk of bias item for each included study.

**Figure 3:**
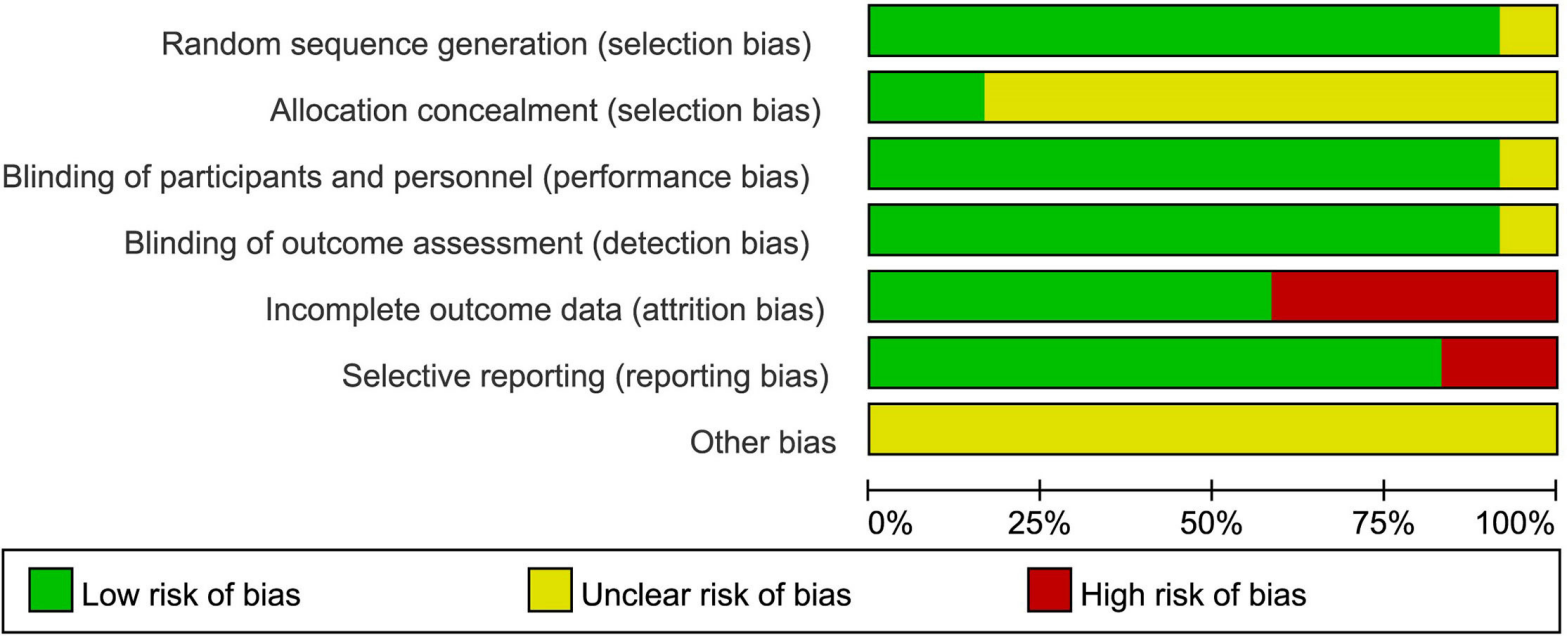
Risk of bias graph: the risks of bias of included studies. Based on the Cochrane's handbook.

The risk of bias in the included studies was evaluated using the criteria described in the Cochrane Handbook. The results were as illustrated in Figure 2, with all included studies using randomized groups. Nine studies described adequate sequence randomization based on random sequences generated using random number tables or computer programs. Two studies reported allocation procedures with adequate concealment. Further, most studies were double-blind to both subjects and evaluators. Therefore, all included studies were considered to have a mild risk of bias (Figure 3).

### Effects of rTMS on MCI

**Figure 4:**
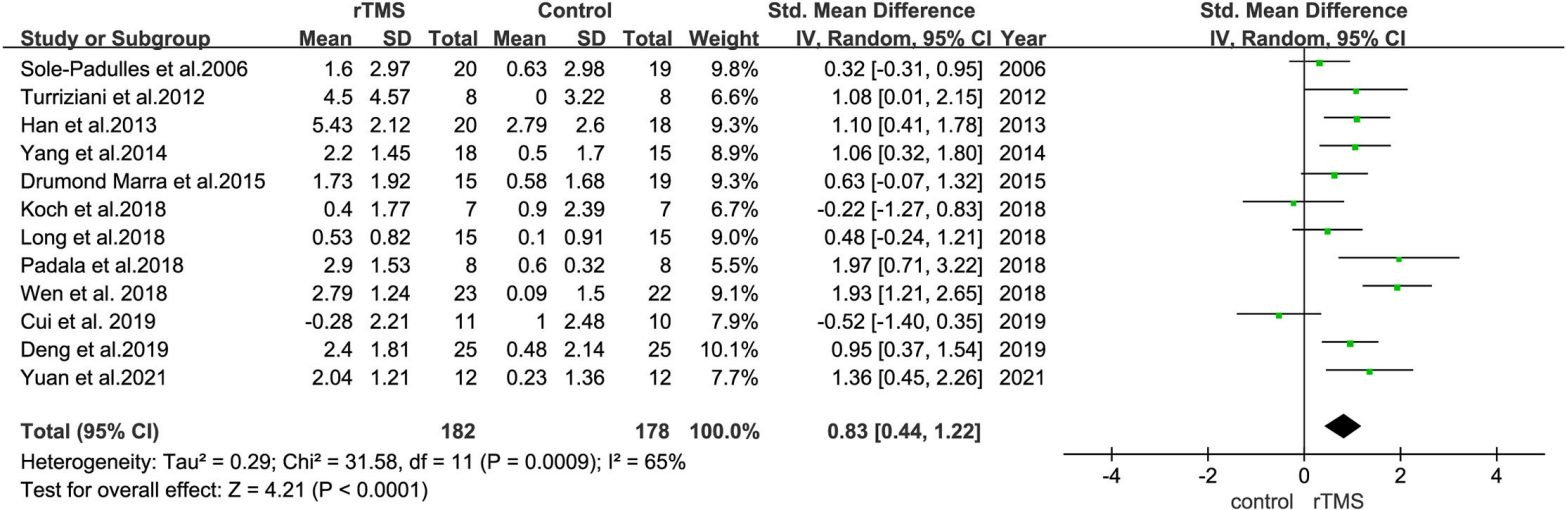
A forest plot representing the overall outcome of rTMS effect on MCI. Mean differences in the effect of rTMS on the overall outcome of MCI with 95% CI.

**Figure 5:**
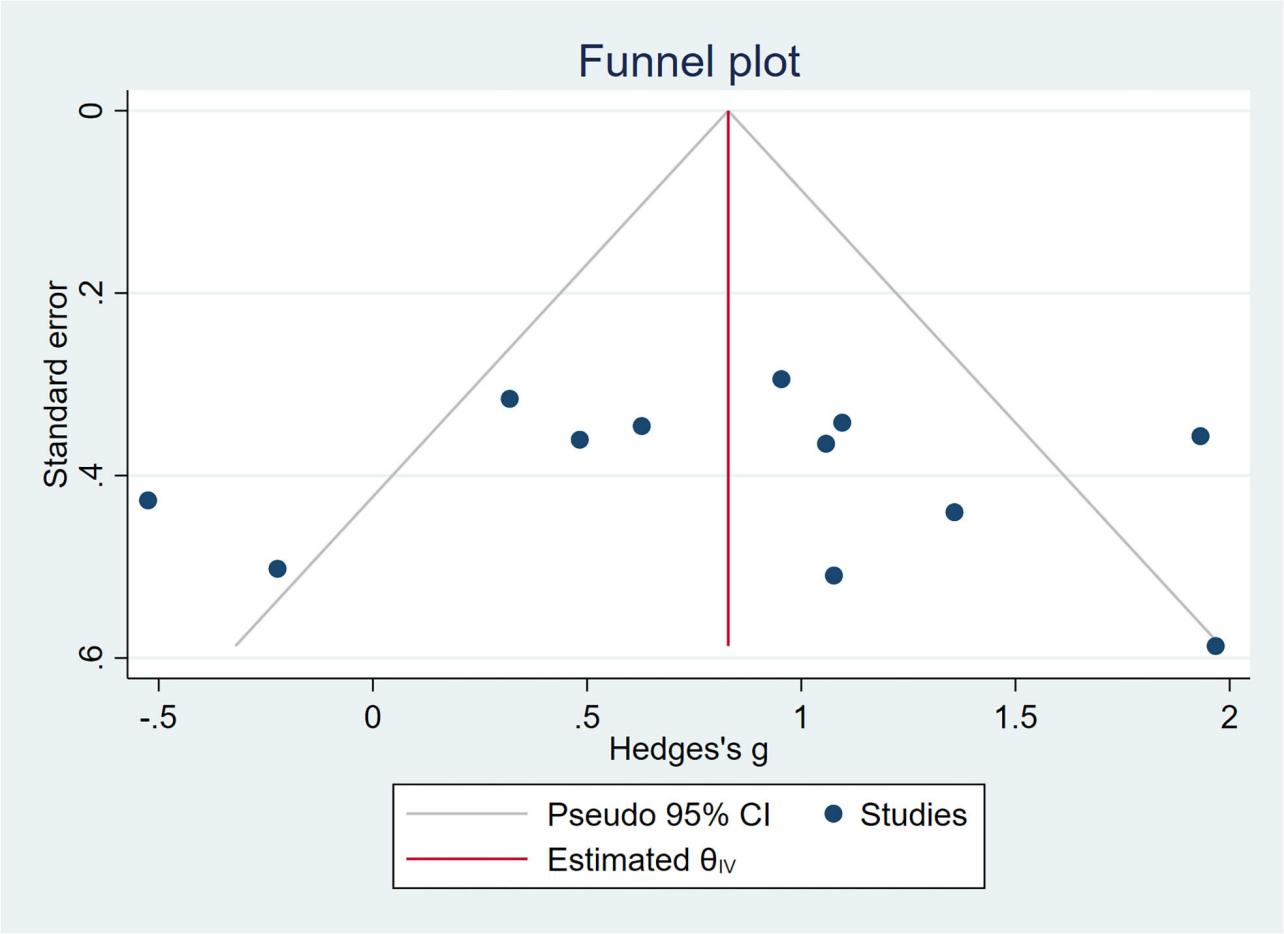
A funnel plot showing publication bias among included studies. The funnel plot was plotted with SMD on the X-axis and the standard error on the Y-axis.

All outcome indicators used for the evaluation of cognitive function in all trials were combined. A random-effects model was used due to the considerable variability in the methods used to evaluate outcome indicators. Pooled results showed that rTMS improved cognitive function significantly among the participants and rTMS treatment exerted a significant effect on cognition in patients with MCI (SMD = 0.83, 95% CI = 0.44–1.22), with moderate between-study heterogeneity (*I*^2^ = 65%, Figure 4). Subgroup analyses were performed to determine variables that may influence cognitive function outcomes. Funnel plots were constructed because >10 studies were included in the present study (Figure 5).

### Subgroup Analyses of the Effects of rTMS on Global Cognition

**Figure 6:**
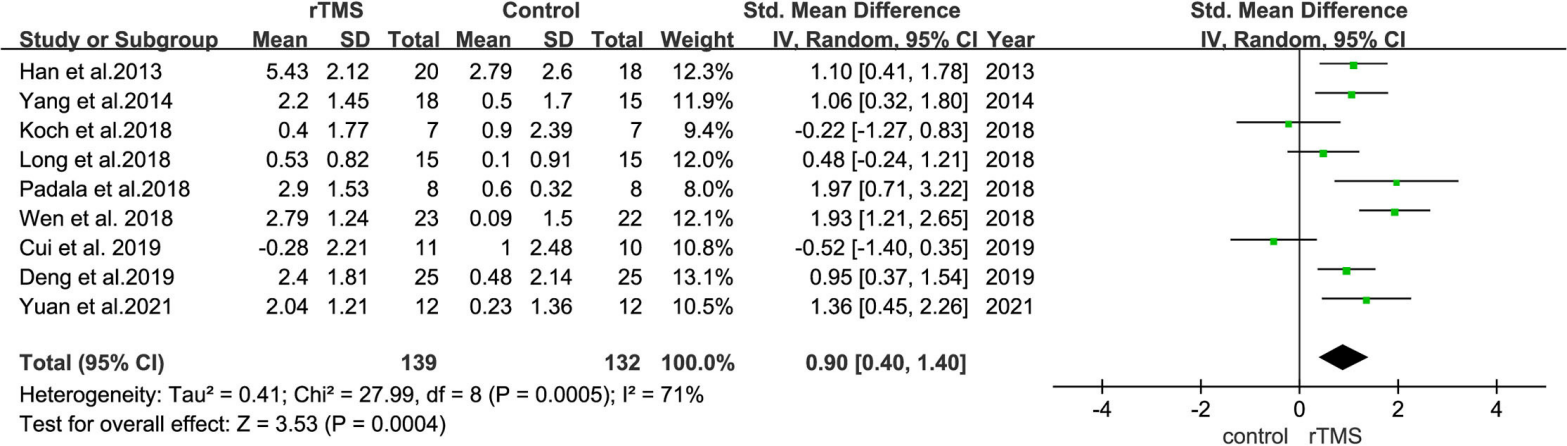
A forest plot displaying the effect of rTMS stimulation on the overall cognitive function in MCI. Mean differences in the effect of rTMS on the overall cognitive function in patients with MCI with 95% CI.

Nine studies evaluated the effect of rTMS on the overall cognition of 271 patients with MCI. It was found that the heterogeneity of included studies was high (*I*^2^ = 71%, *p* = 0.0004), and the meta-analysis was performed using a random-effects model. Sensitivity analyses were also performed, and one study with a low SD (Cui et al., 2019), which did not significantly influence the combined SMD was omitted. The combined results showed that overall cognitive function was better in the rTMS group than in controls (SMD = 0.90, 95% CI = 0.40–1.40, *p* = 0.0004) (Figure 6).

### Subgroup Analysis of the Effect of rTMS on Memory

**Figure 7:**
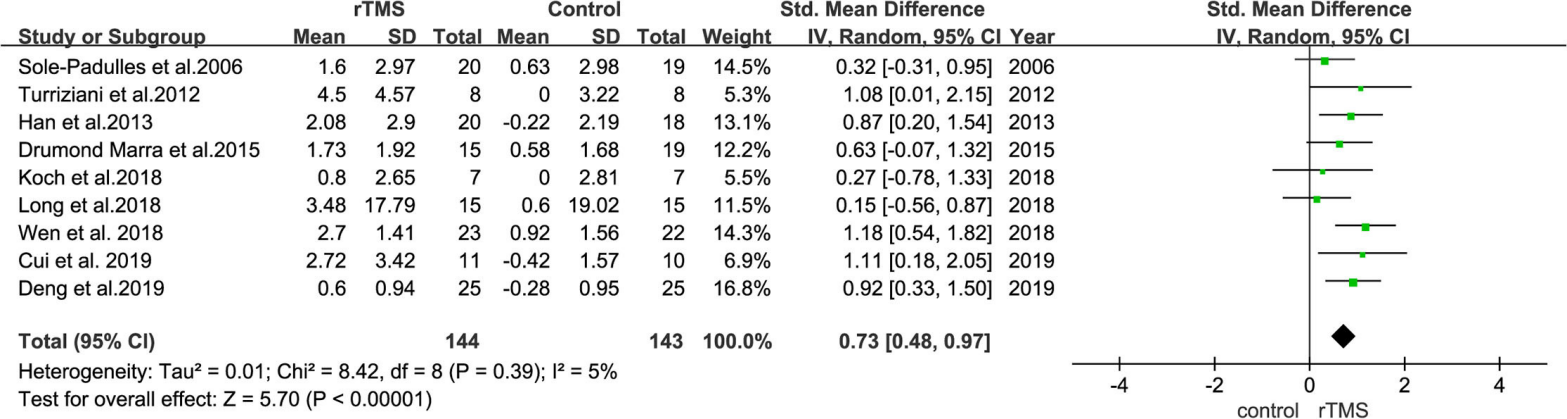
A forest plot showing results of subgroup analyses results for memory outcomes. Mean differences in the effect of rTMS on the memory outcomes in patients with MCI with 95% CI.

The effect of rTMS on memory was reported in the outcome indicators of nine studies. Heterogeneity of included studies was low (*I*^2^ = 26%, *p* = 0.24). Results of the meta-analysis revealed that rTMS significantly improved memory function in patient than control interventions (SMD = 0.73, 95% CI = 0.48–0.97, *p* < 0.00001) (Figure 7).

### Subgroup Analysis of the Effect of rTMS on Stimulation Sites

Subgroup analyses were performed based on stimulation sites (single site and multiple sites). Nine studies with 273 subjects involved stimulation at a single site, whereas three studies with 87 subjects involved stimulation at the bilateral hemisphere with multiple sites.

**Figure 8:**
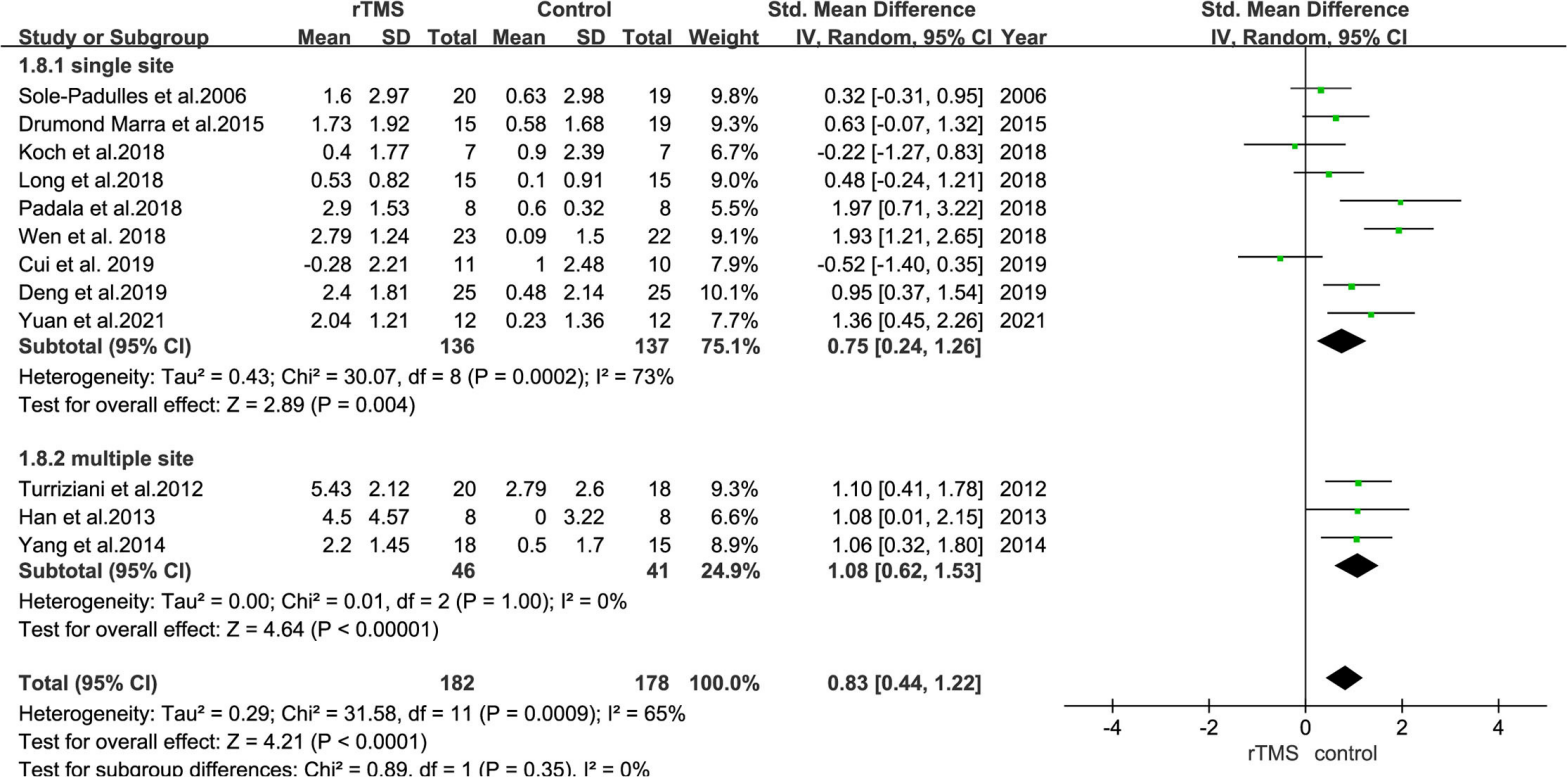
A forest plot showing results of subgroup analyses for stimulation sites (single site vs. multiple sites). Mean differences in stimulation sites subgroup with 95% CI.

The results also revealed that trials involving “single-site” stimulation had an SMD of 0.75 (95% CI = 0.24–1.26). Further, the SMD between trials in bilateral hemispheres was 1.08 (95% CI = 0.62–1.53) (Figure 8).

### Subgroup Analysis of the Effect of the Number of rTMS Stimulation Sessions

The number of stimulation sessions in the studies included ranged from 1 to 40 treatments. Most studies used ≤10 or >10 sessions; therefore, the differences between short-term sessions (≤10 times) and long-term sessions (>10 times) were analyzed.

**Figure 9:**
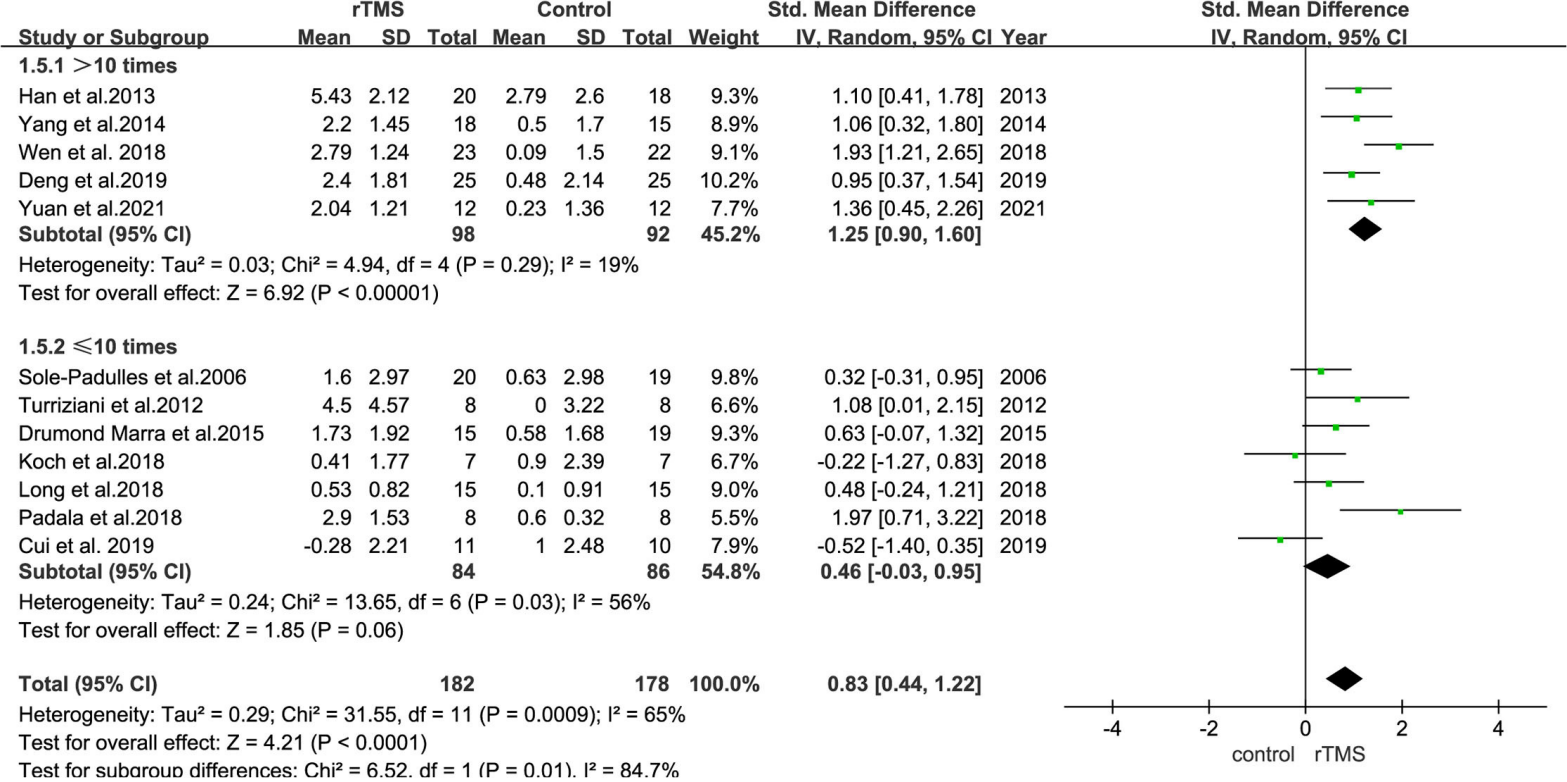
A forest plot showing results of subgroup analyses results for the number of stimulation treatments (>10 vs. ≤10 treatments). Mean differences in stimulation numbers subgroup with 95% CI.

Subgroup analyses revealed that studies with ≤10 stimulation sessions (including 170 participants) had a mean effect size of 0.46 (95% CI = −0.03 to 0.95), whereas those with >10 stimulation sessions (including 190 participants) had a mean effect size of 1.25 (95% CI = 0.90–1.60) (Figure 9). The results suggested that long-term rTMS treatment substantially improved cognitive function more strongly than short-term rTMS treatment.

### Subgroup Analysis of the Safety of rTMS

**Figure 10:**
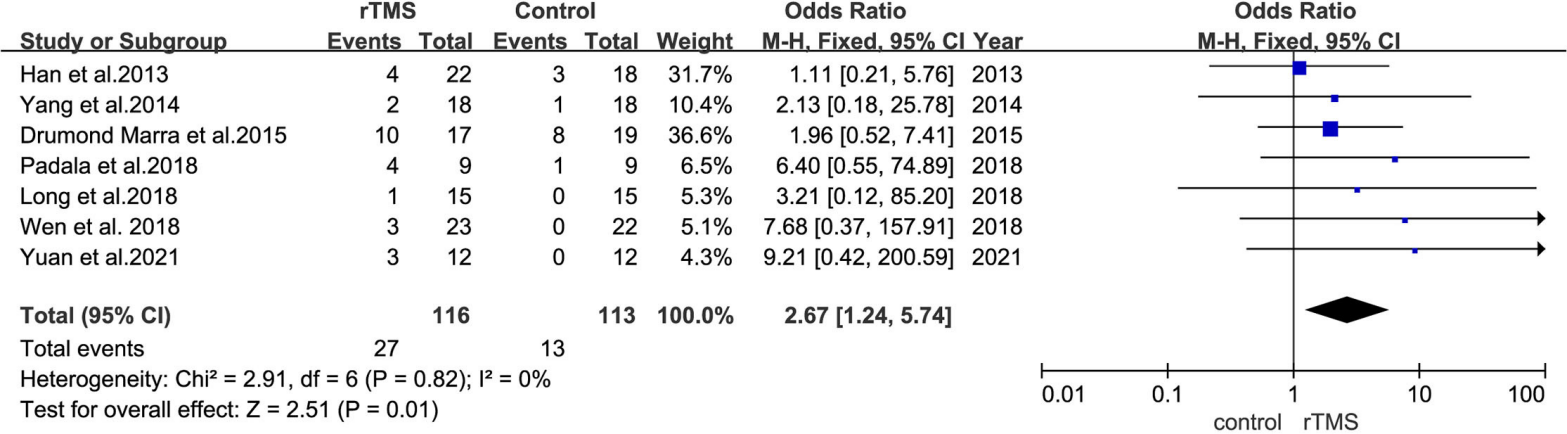
A forest plot displaying results of subgroup analyses results for adverse effects.

Seven studies reported adverse effects. Among them, one study reported a serious adverse effect. One patient presented with two episodes of severe pain after receiving two rTMS treatments and was hence discontinued from the study. All the remaining studies reported mild adverse effects after receiving two rTMS treatments. A total of 27 out of 116 patients in the rTMS group and 13 out of 113 patients in the control group reported discomfort after the treatment. The reported adverse reactions included headache, dizziness, pain in the area of irritation, neck pain, and a burning sensation on the scalp, which resolved rapidly. No heterogeneity was found between studies (*I*^2^ = 0%, *p* = 0.01). Pooled outcomes revealed that the incidence of adverse reactions was higher in the rTMS group than in the control group [risk ratio (RR) = 2.67, 95% CI = 1.24–5.74, *p* = 0.01, seven studies, *n* = 229] (Figure 10).

## Discussion

This meta-analysis included 12 RCT studies with a total of 329 patients with MCI. The outcome indicators in the rTMS group were compared with those of the control group, which received sham rTMS only. Notably, rTMS improved cognitive function in patients with MCI, with a significant effect size (SMD = 0.83).

Results of subgroup analyses revealed that: (1) rTMS improved cognitive function in patients with MCI; (2) the improvement in cognitive function was more pronounced when high-frequency rTMS stimulation was applied to multiple sites, such as bilateral DLPFC; and (3) more than 10 rTMS stimulations were more effective in improving cognition in patients with MCI.

Analysis of rTMS stimulation parameters showed that left or bilateral hemispheric DLPFC sites, high frequency (5–20 Hz), more than 10 stimulations, and stimulation intensity of between 80 and 120% of resting MT were the most effective parameters in improving cognitive function in patients with MCI.

Among the sites reported, the most frequently used stimulation site was DLPFC. An earlier study reported that stimulation of DLPFC reduced depression levels (Downar and Daskalakis, 2013). Subgroup analysis results demonstrated that rTMS stimulation at the DLPFC site improved memory in patients with MCI. Physiologically, DLPFC regulates executive functions, such as working memory and cognitive flexibility (Blumenfeld and Ranganath, 2007; Blumenfeld et al., 2011). Application of rTMS stimulation to the DLPFC locus lessened the symptoms of patients with depression and MCI or AD. According to Chou et al. (2020), the possible explanation for this phenomenon is that the effect of rTMS on cognition may be an indirect effect of improved emotion. DLPFC is highly connected with other regions and is one of the most important regions in the central executive network (CEN). For example, stimulation of DLPFC with high-frequency rTMS can regulate memory-related and executive control-related functions, hence affecting properties of ventral attention network (VAN), CEN, among other networks (Kumar et al., 2017). This indicates that rTMS can improve working memory, perception, and emotions in patients by regulating the brain network (Fox et al., 2014). However, further studies should be conducted to validate the benefits of rTMS on the emotion and cognition function in patients with MCI.

None of the studies included in this analysis compared the outcomes associated with stimulation at different sites. Considering the different neurodegenerative and cognitive impairment characteristics of MCI and AD, the ideal location for rTMS stimulation remains unclear (Heath et al., 2018). Studies with larger sample sizes are required to determine the best stimulation targets for rTMS that yield optimal cognition improvement.

A randomized crossover trial conducted by Koch et al. (2018) reported that rTMS stimulation at the precuneus region medial to the parietal lobe improved episodic memory as determined using the delayed recall scores of the Rey Auditory Verbal Learning Test. However, the scores of other cognitive domains were not improved. In one of the studies, researchers applied rTMS to two separate locations (Taylor et al., 2019), DLPFC and the lateral parietal cortex (LPC). Taylor et al. hypothesized that stimulation of the LPC site might differentially improve memory retrieval and retention, an important cognitive domain of aMCI (unpublished data). A meta-analysis performed by Liu et al. (2019) suggested that DLPFC, inferior frontal gyrus, and temporoparietal junction should be recommended as ideal locations for non-invasive brain stimulation to treat MCI. In addition, the super temporal gyrus (Anderkova et al., 2015), medial superior frontal gyrus (Gogulski et al., 2017), and right inferior occipital gyrus (Vidal-Piñeiro et al., 2014) are also potential brain stimulation sites.

This meta-analysis indicated that dual-locus stimulation of the bilateral cerebral hemispheres produced more pronounced effects than single-locus stimulation of one hemisphere, mainly the left hemisphere. However, these results were not entirely consistent with a previous meta-analysis (Liao et al., 2015). In their study involving 94 patients with mild to moderate AD, Liao et al. found that right-sided or bilateral DLPFC stimulation was superior to single left-sided DLPFC stimulation. This inconsistency with our results might be due to: (1) the meta-analysis by Liao et al. (2015) was published in 2015, and the latest study included was published in 2013, which is relatively old data; (2) the meta-analysis by Liao et al. (2015) only included three studies in which stimulation was performed on the left side of the cortex.

MCI and AD involve functional disturbance or disconnection affecting various brain networks, especially between the frontal lobes, the cingulate cortex, posterior parietal (including precuneus), and temporal regions (Li et al., 2015; Boublay et al., 2016). Paradoxically, treatment strategies using multiple-site stimulation seem no better than those using single-site stimulation (Alcalá-Lozano et al., 2018). For instance, RCTs conducted by Drumond Marra et al. (2015) and Wu et al. (2015) found no significant difference in cognitive improvement between patients stimulated on the left DLPFC only and those stimulated on multiple functional areas. There is evidence that stimulation of a network hub affects the entire cortical and subcortical structures in the network (Fox et al., 2014). Results of this meta-analysis should be interpreted with caution due to the small number of studies included in the analyses of bilateral hemisphere stimulation and multiple site stimulation. Further studies are required to clarify this question.

Subgroup analyses based on the number of stimulation revealed that >10 rTMS treatments were more effective in improving cognition in patients with MCI than ≤10 rTMS treatments. This result is consistent with findings from previous meta-analyses regarding the effects of rTMS on AD (Lin et al., 2019; Chou et al., 2020; Wang et al., 2020), suggesting that long-term rTMS stimulation has better effects. Indeed, previous studies have also demonstrated that long-term rTMS stimulation in patients with neurodegenerative diseases, such as AD and Parkinson's disease, produced good long-term therapeutic effects (Luber and Lisanby, 2014; Chou et al., 2015; Hsu et al., 2015).

The duration of recognition improvement using rTMS is still unclear. In most studies, the efficacy of rTMS was determined at the end of stimulation, and no long-term follow-up was carried out due to the large number of complex factors that hinder effective follow-up. A few studies show that the duration of improvement varies greatly, from 2 to 10 months in AD patients (Cotelli et al., 2011; Rabey and Dobronevsky, 2016). In our study, four studies showed that the duration of improvement ranged from 4 to 8 weeks (Drumond Marra et al., 2015; Wen et al., 2018; Cui et al., 2019; Yuan et al., 2021).

Overall, this meta-analysis reveals that rTMS is a safe and effective non-invasive neural modulation tool in MCI, although it is associated with several adverse effects (Table 2). For instance, one participant withdrew from the study due to intolerable headache (Padala et al., 2018). However, other reported adverse reactions were mild and disappeared within a short period. The most common adverse effects associated with rTMS were mild headaches and dizziness.

**Table 2:** Adverse events reported in studies included in the meta-analysis.

**References**	**Adverse effects in the intervention group**	**Adverse reactions in the control group**	**Withdrawal due to adverse reactions caused by rTMS**
Solé-Padullés et al. (2006)	NR	NR	0
Turriziani et al. (2012)	NR	NR	0
Han et al. (2013)	4 transient headache or dizziness	3 transient headache or dizziness	0
Yang et al. (2014)	2 slight dizziness or scalp pain	1 slight dizziness or scalp pain	0
Drumond Marra et al. (2015)	10 headache, 9 scalp pain	5 headache, 1 neck pain, 2 scalp pain, 1 burning scalp	0
Koch et al. (2018)	NR	NR	0
Padala et al. (2018)	14 treatment site discomfort	2 neck discomfort	1 severe pain
Wen et al. (2018)	3 mild headaches	0	0
Long et al. (2018)	1 mild dizziness	0	0
Cui et al. (2019)	0	0	0
Deng et al. (2019)	0	0	0
Yuan et al. (2021)	3 mild headaches at the beginning of treatment.	0	0

*NR, not reported in the article*.

### Limitations

This current meta-analysis had some limitations. First, the number of studies included and sample size were small. Second, the duration of rTMS effects was not assessed. Third, there was substantial heterogeneity among the stimulation parameters used. The number of studies that applied stimulation at loci other than DLPFC was small, which may influence this study's results. Fourth, this study only evaluated cognition. Therefore, future studies should analyze affective change. Fifth, only one of the studies included used low-frequency rTMS. Thus, the study did not compare the effects of high-frequency rTMS and low-frequency rTMS stimulation. Finally, the sample size used in subgroup analyses was relatively small; hence, the results should be interpreted with caution. Therefore, further RCTs should be conducted to validate the present findings.

## Data Availability Statement

The raw data supporting the conclusions of this article will be made available by the authors, without undue reservation.

## Author Contributions

XZ and XL were responsible for the literature screening and data extraction. XL and CC were responsible for the risk of bias assessment. XZ was responsible for statistical analysis and writing up of this article. HR and YG were responsible for planning and guidance on this study. All authors contributed to the proofreading of this article and approved the submitted version of the manuscript for publication.

## Funding

This study was supported by the Shenzhen Science and Technology Innovation Commission (Nos. KCXFZ20201221173400001 and KCXFZ20201221173411032), the Natural Science Fund of Guangdong Province (No. 2021A1515010983), and Shenzhen Key Medical Discipline Construction Fund (No. SZXK005).

## Conflict of Interest

The authors declare that the research was conducted in the absence of any commercial or financial relationships that could be construed as a potential conflict of interest.

## Publisher's Note

All claims expressed in this article are solely those of the authors and do not necessarily represent those of their affiliated organizations, or those of the publisher, the editors and the reviewers. Any product that may be evaluated in this article, or claim that may be made by its manufacturer, is not guaranteed or endorsed by the publisher.
